# Little Ice Age climatic erraticism as an analogue for future enhanced hydroclimatic variability across the American Southwest

**DOI:** 10.1371/journal.pone.0186282

**Published:** 2017-10-16

**Authors:** Julie Loisel, Glen M. MacDonald, Marcus J. Thomson

**Affiliations:** 1 Department of Geography, Texas A&M University, Eller O&M Building, College Station TX; 2 Institute of the Environment and Sustainability, University of California Los Angeles, La Kretz Hall, Los Angeles CA; 3 Department of Geography, University of California Los Angeles, Bunche Hall, Los Angeles CA; Chinese Academy of Sciences, CHINA

## Abstract

The American Southwest has experienced a series of severe droughts interspersed with strong wet episodes over the past decades, prompting questions about future climate patterns and potential intensification of weather disruptions under warming conditions. Here we show that interannual hydroclimatic variability in this region has displayed a significant level of non-stationarity over the past millennium. Our tree ring-based analysis of past drought indicates that the Little Ice Age (LIA) experienced high interannual hydroclimatic variability, similar to projections for the 21^st^ century. This is contrary to the Medieval Climate Anomaly (MCA), which had reduced variability and therefore may be misleading as an analog for 21^st^ century warming, notwithstanding its warm (and arid) conditions. Given past non-stationarity, and particularly erratic LIA, a ‘warm LIA’ climate scenario for the coming century that combines high precipitation variability (similar to LIA conditions) with warm and dry conditions (similar to MCA conditions) represents a plausible situation that is supported by recent climate simulations. Our comparison of tree ring-based drought analysis and records from the tropical Pacific Ocean suggests that changing variability in El Niño Southern Oscillation (ENSO) explains much of the contrasting variances between the MCA and LIA conditions across the American Southwest. Greater ENSO variability for the 21^st^ century could be induced by a decrease in meridional sea surface temperature gradient caused by increased greenhouse gas concentration, as shown by several recent climate modeling experiments. Overall, these results coupled with the paleo-record suggests that using the erratic LIA conditions as benchmarks for past hydroclimatic variability can be useful for developing future water-resource management and drought and flood hazard mitigation strategies in the Southwest.

## Introduction

Over the past century, water management has relied on the principle of stationarity, which assumes that historical hydroclimatic variations provide an envelope within which future conditions are expected. However, some areas of the American Southwest will likely become periodically more arid than the range of observations recorded over the last century [1,2], and thus the assumption of stationarity for forecasting future hydroclimatic variability is not justified [3]. Several studies suggest that, along with warmer mean temperatures, climate variability is likely to increase [4,5]. As climate variability increases, the frequency of extreme events is likely to increase as well [4], with direct consequences in the Southwest. For example, an increase in the frequency or magnitude of floods and droughts could lead to yield reductions, crop damage, and crop failure [6]. Warmer and more variable conditions would also impact ecological systems, with one of the most important aspects being fire regimes [7,8] and populations of vulnerable species [9]. Enhanced variability in precipitation promotes fire in many wildland systems [10–13]. Therefore, identifying strategies for water-resource management under changing climate variability, and not just changes in the mean state, is necessary [14].

Instrumental records have shown that hydroclimatic variability across the American Southwest is mostly structured around cool-season precipitation regimes, with a few winter storms typically contributing a disproportionately large amount of the annual precipitation across this region [15]. That is particularly the case in California, where decadal precipitation variance is typically equivalent to 20–50% of mean annual averages, mostly because of changes in precipitation received between November and March [16–17]. As a result, small surpluses or deficits in the number of precipitation events translate into relatively large hydroclimatic swings from wet to dry years. In addition to providing a considerable fraction of the annual total amount, winter precipitation tends to remain in the ‘terrestrial’ hydrological cycle (i.e., part of stream flow) much longer than the summer fraction [17], making the cool-season precipitation regime particularly important for natural processes and human consumption. The large interannual to decadal hydroclimatic variability in winter precipitation is highly influenced by sea surface temperature (SST) anomalies in the tropical Pacific Ocean and associated changes in large-scale atmospheric circulation patterns [16]. In general, cool SSTs in the eastern tropical Pacific (La Niña conditions) tend to induce arid conditions in the Southwest, whereas warmer SSTs (El Niño conditions) are associated with relatively wet conditions [18,19].

**Fig 1:**
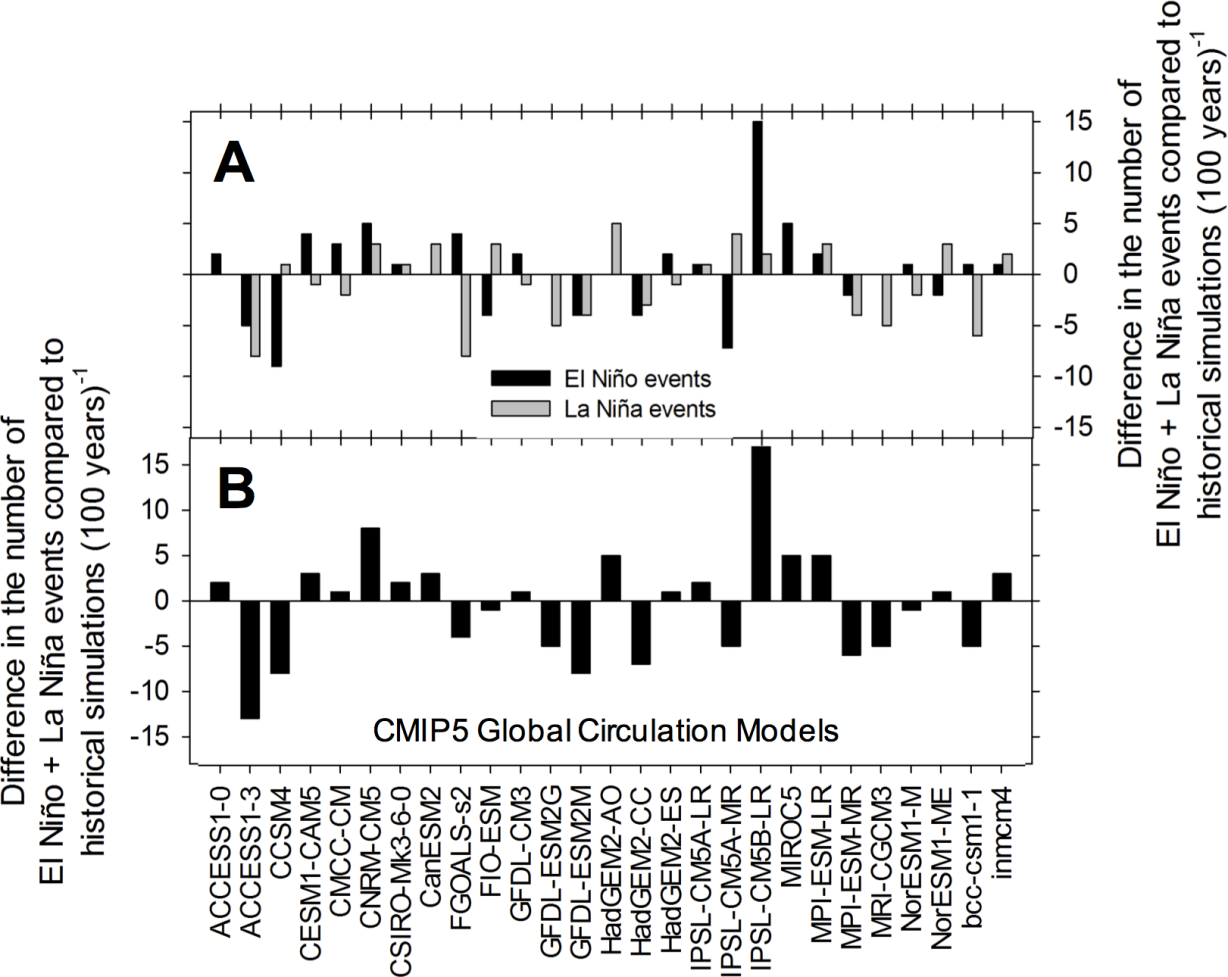
Predicted number of El Niño and La Niña events during the 21^st^ century compared to historical simulations. Data are based on 27 CMIP5 models and using the RCP8.5 scenario. The overall sum of predicted El Niño events is +10, while that of La Niñas is -19. Overall, 70% of the models indicate either no change or an increase in the number of El Niño events for the coming century; this figure goes down to 52% for La Niñas (data from 21).

**Fig 2:**
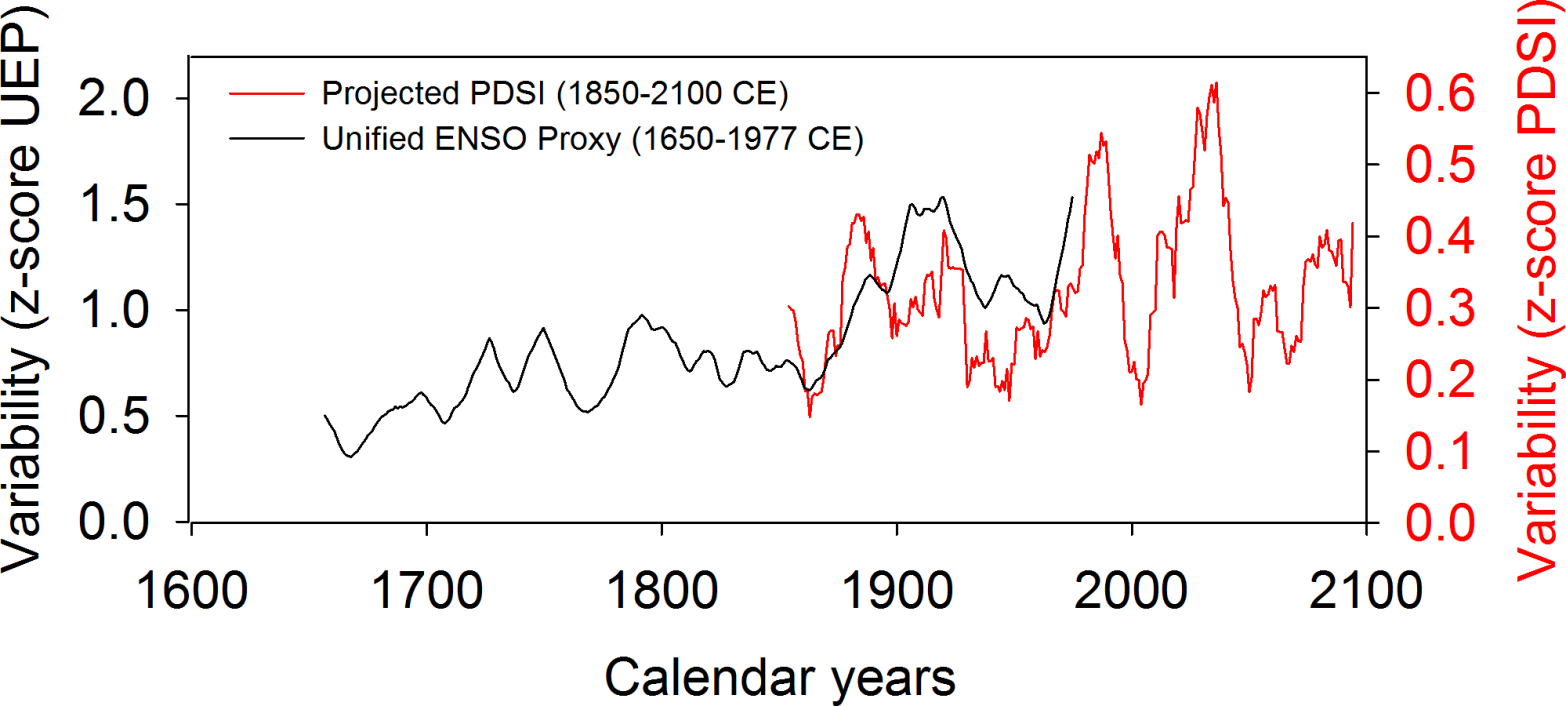
Unified ENSO proxy (UEP) variability compared to projected PDSI variability across the American Southwest in the coming century. The UEP (in black) combines ENSO and PDO/IPO Pacific climate variability and is based on 10 commonly used ENSO proxies that were consolidated via Principal Component Analysis to capture the joint features of these reconstructions (data from 23). The multimodel mean summer (JJA) PDSI variability over the American Southwest for 1850–2100 (in red) is based on 17 CMIP5 model projections and using the RCP 8.5 emissions scenario (data from 24).

Temporal precipitation variability in the Southwest may increase significantly at the decadal and multi-decadal scale over the 21^st^ century. Several climate predictions for future impacts of increasing radiative forcing suggest warming in the eastern Pacific and a more variable ENSO system, with ~70% chance of stronger and/or more frequent El Niño conditions, and a ~50% chance of increased frequency in La Niñas (Fig 1; [20,21]). Such potential changes in variability are in agreement with instrumental records and paleoclimate reconstructions, which show that the magnitude and trend of hydroclimatic variability has not been constant in the Southwest during the Common Era (C.E.). As such, recent studies looking back at the past 30 years of data show a decadal modulation of El Niño Southern Oscillation (ENSO) stability, with the past decade characterized by higher-frequency and lower-amplitude El Niños than the previous ones [22]. In addition, the unified ENSO proxy (UEP) time series, which combines information from 10 globally distributed ENSO reconstructions, displays a rising trend toward enhanced ENSO variance since 1650 CE (Fig 2; [23]).

On land, multimodel mean PDSI time-series projections for the coming century indicate that drought risk could reach an all-time high during the late 21^st^ century, with unprecedented drought conditions that might exceed those of the MCA [2,24]. These modeled PDSI values are also characterized by high variance [Fig 2], reinforcing the hypothesis that the coming century will likely be faced with high hydroclimatic variability that could be linked to ENSO dynamics. These results remain speculative however, as there are large differences in PDSI projections between models [Fig 2].

The Medieval Climate Anomaly (MCA, ~950–1400 CE) is often used as an analog for 21^st^ century hydroclimate because it represents a warm (and arid) period. The MCA appears related to general surface warming in the Northern Hemisphere, prolonged La Niña conditions [18, 25–28], and a persistent positive North Atlantic Oscillation mode [29]. It has been referred to as a stable time interval with ‘quiet’ conditions in regards to low perturbation by external radiative forcing [30]. In this study, we demonstrate that the Little Ice Age (LIA, ~1400–1850 CE) might be more representative of future hydroclimatic variability than the conditions during the MCA megadroughts for the American Southwest, and thus provide a useful scenario for development of future water-resource management and drought and flood hazard mitigation strategies.

## Materials and methods

### Paleohydrological reconstructions and model simulations

**Fig 3:**
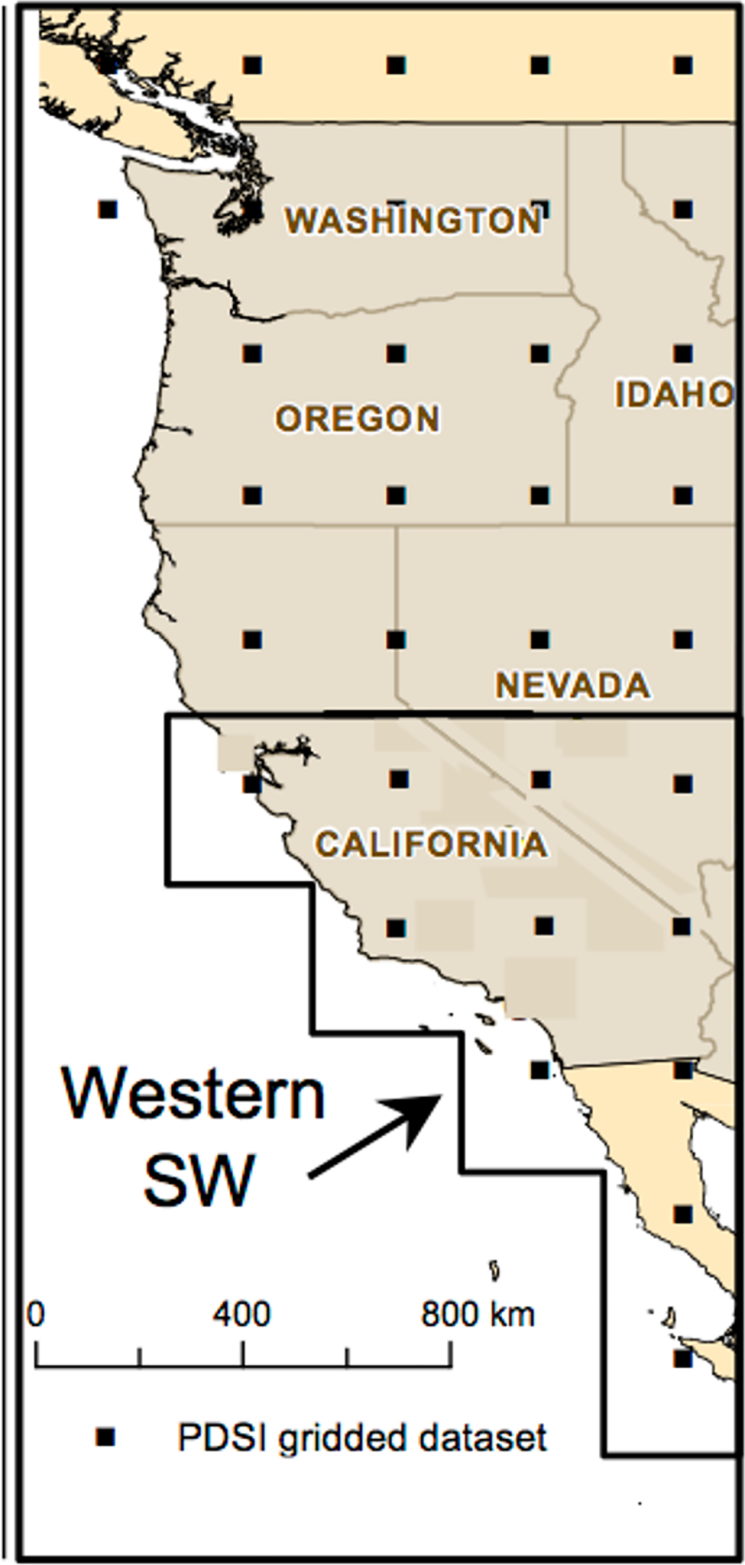
Study region. North American Drought Atlas (NADA) gridded dataset (http://www.ncdc.noaa.gov/paleo/pdsi.html; https://iridl.ldeo.columbia.edu/SOURCES/.LDEO/.TRL/.NADAv2a-2008/PDSI/datafiles.html) used in this study. The bold black line delineates the western portion of the American Southwest.

Tree ring-derived Palmer Drought Severity Index (PDSI) time series have been widely used to estimate the spatial extent, duration, timing, and intensity of droughts from the past millennium across the American Southwest [31–37], but the amplitude and variance of hydroclimatic variability have received significantly less attention (but see [38]). We used PDSI values from the North American Drought Atlas (NADA, version 2a) to detect changes in aridity variability and periodicity for the Southwest over the past millennium. The NADA consists of a network of 286 gridded data points (2.5° x 2.5°) covering North America that is based on annually resolved tree ring chronologies [39,40]. Information related to reconstruction methods can be found in [39]. Grid points that cover the western Southwest (32.5–37.5°N, 115–122.5°W, n = 9 grid cells) were selected on the basis of their climatology [Fig 3]. Annual PDSI values for these study regions were obtained from 950 to 2006 CE.

The El Junco diatom index record from the Galápagos Islands (0°54'S, 89°29'W, 43) and the fossil-coral oxygen isotopic records from Palmyra Island (6°N, 162°W, 44) constitute ENSO-sensitive proxy records from the equatorial Pacific against which we compared our results. Both records were resampled at an annual time step prior to statistical analysis. Information related to the study sites, reconstruction methods, and chronologies can be found in the original publications [41,42].

Winter precipitation data for the past millennium were obtained from the Community Earth System Model’s Last Millennium Ensemble Project (CESM LME) [43]. CESM LME generated ten ensemble members with ‘full’ forcing, which consists of transient solar output, volcanic activity, land use, greenhouse gases, and orbital dynamics for the complete 850–2005 time series, as well as ozone-aerosol forcing for the 1850–2005 series used for bias correction. The ensemble member approach is commonly used to approximate a measure of uncertainty in modeled results. We analyzed months November through February to represent ENSO influence on the regional precipitation regime. We used ensemble members 2 to 5, which consist of coupled ocean-atmosphere climate models; outputs represent a range of probable monthly precipitation values generated by CESM. Precipitation data were bias corrected using NOAA’s CPC US unified daily precipitation data provided by NOAA/OAR/ESRL PSD [*http://www.esrl.noaa.gov.psd/*]. Grid cells over the Pacific were excluded from the analysis.

### Statistical analyses

Variability measures applied to NADA PDSI, El Junco diatoms, Palmyra isotopes, and modeled precipitation from CESM were developed from the original, raw time series. Each time series was filtered (10-year high-pass) using AnalySeries 2.0.4.2 [44] to preserve variability in the ENSO band at 2 to 8 years [45]. Variance was then computed as the 10-year running standard deviation of each filtered time series [38] and rescaled to z-scores.

Student’s t-tests were used to determine whether or not the MCA (950–1400 CE), LIA (1400–1850 CE), and RW (1850–2006) datasets were significantly different from each other in terms of their variance. Lastly, a bias-corrected wavelet analysis was used to identify dominant periodicities in the unfiltered mean PDSI time series between the MCA, LIA, and RW [46]. Tolerance level for significance of dominant frequencies against red-noise background spectrum was set at 0.95 [47].

## Results

### Paleohydrological reconstructions

**Fig 4:**
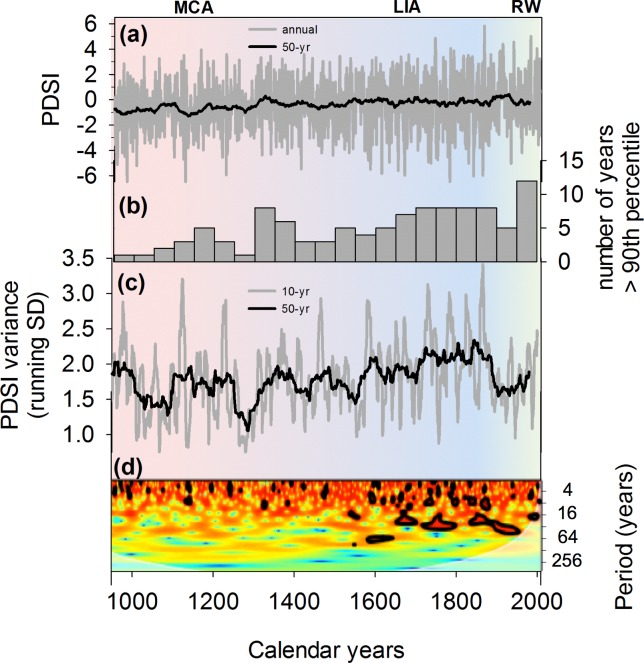
Palmer Drought Severity Index (PDSI) variability in the American Southwest over the past millennia. The annual time series (a) displays greater variability during the LIA than the MCA, as indicated by the number of years during which variability is above the 90^th^ percentile (b). The time series was filtered using a 10-year high-pass to compute a time series of PDSI variance (c) on the basis of 10-year and 50-year running standard deviations (SD). A wavelet analysis shows the evolution of the power spectrum of tree-ring derived PDSI over the past millennia (d). The black contours are the 10% significance regions, using a red-noise background spectrum [46].

Analyses performed on the NADA PDSI time series show a number of important and statistically significant differences in hydroclimatic variability between the MCA, LIA, and RW (Table 1, Fig 4). Variance in PDSI amplitude is significantly lower during the MCA than during the LIA and the RW (Tukey’s LSD: p < 0.001). Likewise, mean running variance is overall lower (p < 0.001) during the MCA than during the LIA and the RW (Fig 4C). The evolution of change in drought amplitude from the MCA to the LIA continues during the RW, with LIA variance being indistinguishable from that recorded during the RW (p = 0.22). In addition, the evolution of change in drought amplitude clearly shows greatest variance during the LIA, with roughly half (53%) of years for which PDSI’s mean running variance stays within the highest 10th percentile (Fig 4B).

**Table 1:** Moments of the distribution of filtered paleoecological records and climate simulations.

dataset	time period	mean of running variance	standard dev. of running variance
Western Southwest PDSI	MCA	1.66***	0.49
(CA-NV)	LIA	1.91***	0.47
	RW	1.86***^1^	0.46
El Junco	MCA	0.08***	0.03
	LIA	0.12***	0.05
	RW	0.18**^2^	0.05
Palmyra	MCA	0.09**	0.02
	LIA	0.10**	0.03
	RW	0.12**	0.02
Winter P (NDJF), CESM	MCA	0.04	1.17
em002	LIA	-0.04	0.89
	RW	-0.10	0.73
Winter P (NDJF), CESM	MCA	0.13***	1.67
em003	LIA	-0.12***	0.91
	RW	-0.02	1.01
Winter P (NDJF), CESM	MCA	-0.02	0.99
em004	LIA	0.01	1.02
	RW	0.02	0.95
Winter P (NDJF), CESM	MCA	0.13**	0.99
em005	LIA	-0.05**	1.02
	RW	-0.24**	0.91
Winter P (NDJF), CESM	MCA	-0.13	0.99
averaged em002 to em005	LIA	-0.17	1.02
	RW	0.89***^3^	0.91

*Statistical significance at p < 0.1.

**Statistical significance at p < 0.05.

***Statistical significance at p < 0.01.

^1^The RW is statistically different from the MCA (p < 0.01), but indistinguishable from the LIA (p = 0.22).

^2^The RW time series from El Junco was not used in the statistical analysis as it only contains 5 data points.

^3^The RW is statistically different from the MCA and the LIA (p < 0.05).

A wavelet analysis reveals changes in relative domains of variability over time, with a higher concentration of power in the interannual and decadal bands during the LIA that is much subdued during the MCA (Fig 4D). This shift in quasi-periodic variance confirms the presence of a change in the dominant frequencies of variability between the MCA and LIA boundary. An increase in power of the higher frequency ENSO band of 3 to 8 years was detected, especially after 1550 CE. These shifts in PDSI periodicity combined with a general increase in variance are concomitant with the transition from the MCA to the LIA.

**Fig 5:**
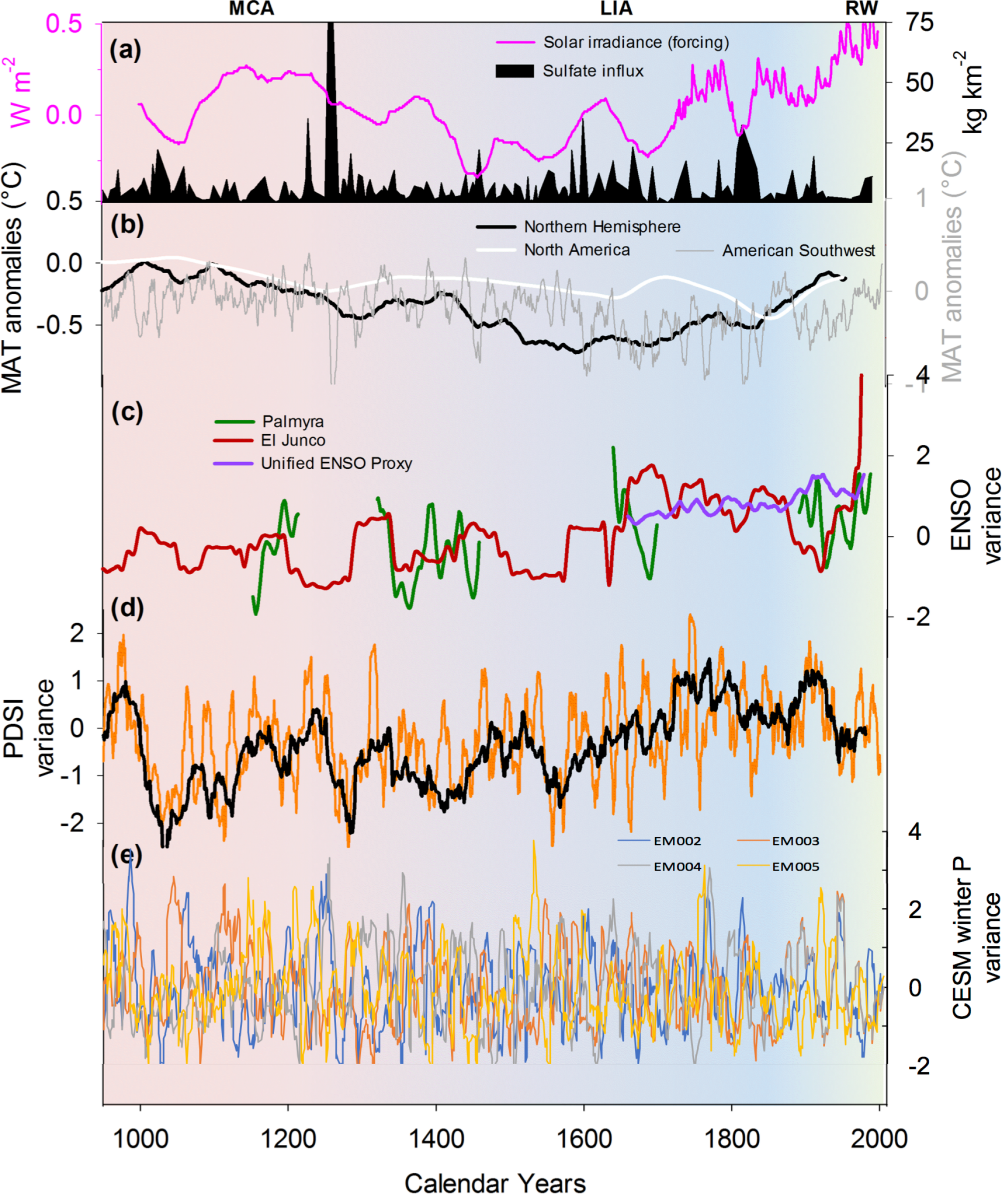
Factors affecting and reflecting hydrologic variability in the American Southwest over the past millennia. Global solar irradiance reconstruction [48–50] and ice-core based sulfate (SO_4_) influx in the Northern Hemisphere [51] from volcanic activity (a); mean annual temperature (MAT) reconstructions for the Northern Hemisphere [52], North America [29], and the American Southwest* expressed as anomalies based on 1961–1990 temperature averages (b); changes in ENSO-related variability based on El Junco diatom record [41], oxygen isotopes records from Palmyra [42], and the unified ENSO proxy [UEP; 23] (c); changes in PDSI variability for the American Southwest (d), and changes in winter precipitation variability as simulated by CESM model ensembles 2 to 5 [43]. *Data for the American Southwest temperature reconstruction is from PRISM temperature data from CMIP5 [43].

Evidence of similar changes in variance and variability are clear from ENSO-sensitive proxy records from the equatorial Pacific (Fig 5, Table 1). The fossil-coral oxygen isotopic records (δ^18^O) from Palmyra Island present similar, and climatologically consistent, changes to the Southwest PDSI variance over time, including decreased mean state of ENSO in the MCA (a cooler eastern equatorial Pacific) and more intense ENSO in the LIA, particularly during the mid-seventeenth century. Variance in δ^18^O amplitude was lower during the MCA than during the LIA and the RW (Table 1). Likewise, mean running variance was lower overall (p < 0.001) during the MCA than during the LIA and the RW. As the Palmyra fossil-coral records are not continuous, these statistics should be used with caution. That said, the continuous diatom record from El Junco in the Galápagos Islands similarly shows enhanced ENSO variability during the LIA when compared to the past millennium (not shown). Noteworthy is the significantly lower variance (p = 0.02) in the diatom index during the MCA when compared to that of the LIA. Likewise, mean running variance was overall lower during the MCA than during the LIA. As there are only five data points characterizing the RW, results were considered inconclusive (Table 1).

### Model simulations

Mean modeled winter precipitation from CESM LME ensemble members 2 to 5 show unsystematic differences in Southwest winter precipitation variability between each other and with our NADA PDSI time series (Table 1, S1 Fig). For instance, mean variance of MCA winter precipitation amounts was more variable than those of the LIA for ensemble members 3 and 5 (p < 0.05). Likewise, mean variance in winter precipitation during the RW was lower than those simulated for the LIA and MCA in ensemble member 5 (p < 0.05). None of the statistical analysis pertaining to interannual variability in ensemble members 2 and 4 returned significant relationships. Averaging across all 4 model ensembles, we find more variable conditions during the RW than the MCA and LIA (p < 0.001), but no difference between the MCA and the LIA (p = 0.46).

### Data interpretation

The evidence presented here shows that the changes observed in PDSI running variance for the Southwest are coeval with changes in ENSO variance observed in other tropical Pacific Ocean records (Fig 5). The linkages between these changes in Southwest hydroclimatic variance and changes in the Pacific are consistent with modeled and observed historical teleconnections between the Pacific and North America [19,25] and, taken together, support a pervasive relationship between the evolution and variability of the ENSO system and the evolution of drought amplitude and variability in the Southwest [38]. These changes in tropical Pacific Ocean SSTs over the past millennium have often been associated with internal variability of the ocean-atmosphere system [19,27,53,54] that may not be accurately represented in current climate models. The latter would explain the lack of coherence in terms of variability between CESM-derived winter precipitation ensemble members and NADA-derived hydroclimatic conditions across the American Southwest. Model error in representing the impact of SST anomalies on land is also possible. Noteworthy is that model variability is reflecting volcanic forcing rather than changes in ENSO variability.

## Discussion

### Climate forcing factors during the past millennium

Radiative forcing due to changes in solar irradiance and volcanic activity were arguably important drivers in the MCA and LIA [Fig 5]. For example, the minima in solar irradiance combined to the increase in explosive volcanism after the 12^th^ century have been proposed as mechanisms capable of explaining the cooler LIA conditions [55–57]. Likewise, the period of relative stability in terms of solar irradiance combined with minimal volcanic activity could have induced the MCA [30]. These changes in external forcing could partly explain the change in variance and periodicity found in our analysis of PDSI between the MCA and LIA [Fig 4]. As future changes in total solar irradiance and volcanic activity remain unknown, they are not included in radiative forcing calculations used for future climate simulations [58]. These forcing factors constitute additional elements that could drive variability in the future, particularly volcanic aerosols following large eruptions [59,60]. In addition, internal variability in the global ocean-atmosphere system as well as stochastic atmospheric variability could lead to additional uncertainty regarding future climate variability [54,61,62]. Our study stresses the importance of those internal connections between tropical Pacific Ocean SSTs, the ENSO system, and the American Southwest hydroclimatic conditions and supports the contention that: (1) internal variability of the ocean-atmosphere system may not be accurately represented in current global climate models, and (2) enhanced variability as a result of these stochastic events should be further considered.

### The impact of ENSO on precipitation variability across the Southwest

Our understanding of ENSO and its connection to background climatic conditions comes largely from the paleoclimate record and model simulations. We know that ENSO was systematically weaker during the early and middle Holocene, probably as a consequence of boreal summer perihelion and associated change in length and timing of seasons [63]. Orbital forcing combined with a waning Laurentide ice sheet thus suppressed ENSO until around 5000 ka (1 ka = 1000 calibrated years before present), after which its behavior emerged from records of the Pacific region [42]. The drivers of change in the ENSO regime over the past millennium differ from those in the mid-Holocene. The former is the result of internal variability and radiative forcing (solar output and volcanic activity) rather than long-term changes in Earth’s orbital geometry. While there is evidence of a persistent relationship between periods of aridity during the mid-Holocene and the MCA, as both are associated with increases in radiation and cooler SST in the eastern Pacific [64], climate simulations suggest that current forcing by increased GHG may produce an opposite oceanic response in the future [65]. Indeed, an increase in GHG could lead to surface warming over the eastern Pacific followed by an expansion of the warm pool. This would result in decreases in both the meridional and the east-west SST gradients. Those conditions could lead to an increase in ENSO amplitude and/or frequency [21,22,65–67]. While we cannot assert with confidence whether this ongoing shift is part of natural ENSO variability or a manifestation of GHG-induced climate change [68], this increase in variance coincides with rising temperatures in the Western Pacific Warm Pool [23,69–71]. As further warming is anticipated in this region of the Pacific and elsewhere, enhanced hydroclimatic variability might be expected across southwestern North America in the coming century. In addition to potential changes in the mean state of the eastern Pacific Ocean, it is therefore important to consider how interannual and decadal-scale variability in the ENSO system, and thus variability in Southwest hydroclimatology, might evolve over the 21^st^ century [68]. We know that ENSO behavior exhibits decadal- to centennial-scale modulation larger than those observed in the instrumental record [72], and that future conditions could, therefore, push the ENSO system beyond the range of return intervals and levels [3].

### A ‘warm LIA’ as a future climate scenario

In the coming century, increasing atmospheric GHG concentration and associated warming could have important hydrological and water resource consequences in the Southwest resulting from mean state changes due to higher evaporation and decreased precipitation [73–75]. This is in addition to the probable role of GHG-induced amplification of the atmospheric waves in the mid- and high-latitudes, which are thought to lead to increased extreme events across the Southwest and beyond [76,77]. However, changing climatic variability is also a concern. In addition to influencing management of regional water supplies and agricultural practices as well as modulating droughts and floods [4,5,78–79], interannual hydroclimatic variability directly affects wildland plant growth, fuel conditions, and fire regime. As such, increasing variability in moisture conditions have been linked to enhanced fires, as wet/dry oscillations promote rapid biomass growth and natural fire suppression (fuel accumulation), followed by subsequent burning [8,10–13]. Increasing drought frequency and warming temperatures (fuel moisture) have also been positively associated with increased wildfire activity, particularly in Western North America [39,80–84]. In the Southwest, it has previously been shown that largest fire years tend to be experienced after a wet-dry sequence [7], and in association with an El Niño-La Niña sequence. A pattern of enhanced fire activity during times of increased variability in ENSO and Southwest hydroclimatic conditions could imply a trajectory towards a more fire-prone Southwest during the 21st century [7,13]. Projected warming and drying in spring and summer combined with earlier snowmelt and more winter rain would likely exacerbate this trend by facilitating fire ignition and diminishing fuel moisture during the dry season [85].

In light of our findings and its implications, we propose a ‘warm LIA’ scenario for the Southwest, which compounds the effects of warmer temperatures with higher hydroclimate variability. Under this previously non-analogue scenario, enhanced drought-prone conditions would be interspersed with flood-prone ones against a background of overall water resource diminishment. Warmer temperatures would alter the rain/snow ratio during the cold season, further increasing the chance for more extreme winter floods and summer droughts [15]. In agreement with this speculation are climate projections suggesting increased flood magnitude in the future across the Southwest, despite reduced mean precipitation amounts [4].

## Conclusion

Forecasting hydroclimatic conditions in the American Southwest requires thorough consideration of regional climate non-stationarity in the higher moments and not just mean state. This region is inherently prone to highly variable precipitation, including episodic droughts as well as rapid snowmelt and severe rainstorms that often lead to flooding. Our results show that hydroclimatic variability in the Southwest has not remained constant over the last millennia, with a shift from low to high variance at the MCA-LIA transition that was accompanied by a change in quasi-periodic variance, from a higher concentration of power in the multi-decadal periodicities during the MCA vs. interannual and decadal periodicities during the LIA. Shifts in variance are corroborated by ENSO-sensitive proxy records from the tropical Pacific, suggesting an interactive relationship between the ENSO system and the evolution of drought amplitude in the Southwest. In line with a potential increase in decadal variability in the ENSO system over the 21^st^ century [86], we argue that LIA variability provides crucial targets in the paleoclimate record against which to scale the importance of future hydroclimatic variability in the American Southwest. This finding does not preclude the importance of Medieval-era droughts as benchmarks to assess the severity of future drought risks [24,25,37]. Rather, we propose the possible development of a ‘warm LIA’ climate scenario for the coming century that combines high precipitation variability (similar to LIA conditions) with warm and dry conditions. These observations further challenge assumptions of climate stationarity and offer new awareness of climate risks for ensuring sustainable water and land management in the Southwest. These observations can also be useful in efforts to understand and reduce model uncertainties related to ENSO behavior and impacts in attempts to model future Southwest climate.

## Supporting information

S1 Fig Winter precipitation variance in the American Southwest for the past millennium, as simulated by CESM LME. We used ensemble members 2 to 5, which consist of coupled ocean-atmosphere climate models; outputs represent a range of probable monthly precipitation values generated by CESM [43].
(PDF)
