# Supplementary figures and images for: Little Ice Age climatic erraticism as an analogue for future enhanced hydroclimatic variability across the American Southwest
Source: PLoS One. 2017 Oct 16;12(10):e0186282. doi: 10.1371/journal.pone.0186282 (PMC5643054; doi:10.1371/journal.pone.0186282)

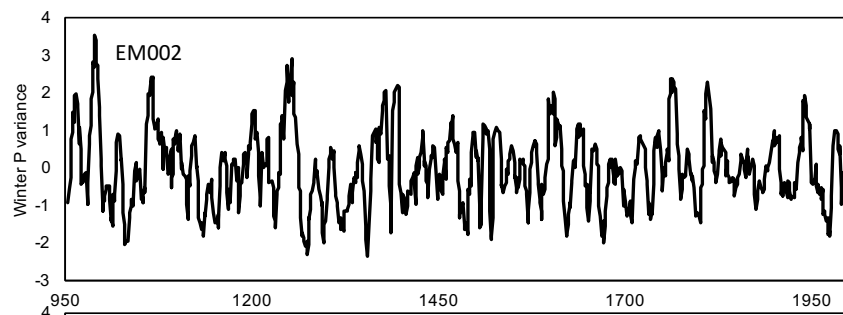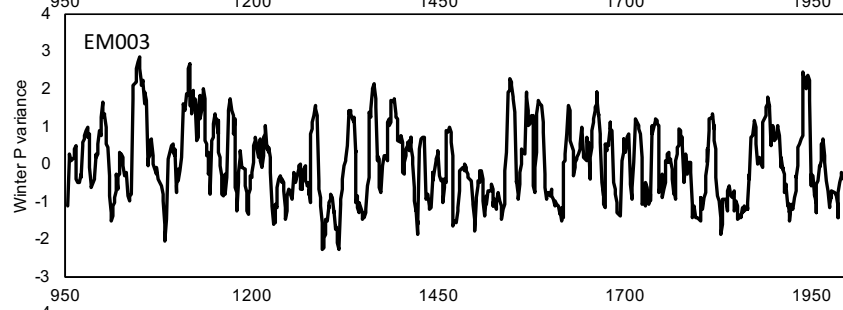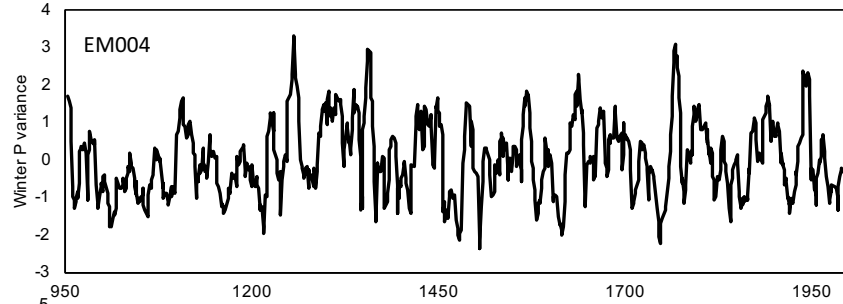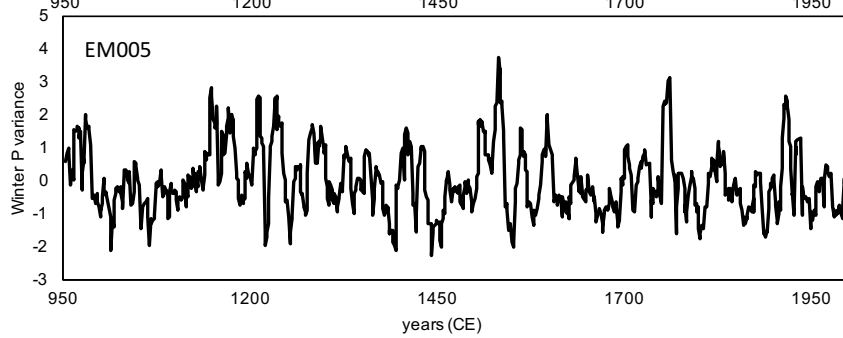

Supplement: S1 Fig — We used ensemble members 2 to 5, which consist of coupled ocean-atmosphere climate models; outputs represent a range of probable monthly precipitation values generated by CESM [43]. (PDF) [file pone.0186282.s001.pdf]
